# Editorial: Strategies to Fight Exercise Intolerance in Neuromuscular Disorders

**DOI:** 10.3389/fphys.2020.00968

**Published:** 2020-08-19

**Authors:** Francesca Lanfranconi, Mauro Marzorati, Lucio Tremolizzo

**Affiliations:** ^1^Monza and Brianza Foundation for Children and Mothers, Monza, Italy; ^2^Institute of Biomedical Technologies, National Research Council, Segrate, Italy; ^3^School of Medicine and Surgery, University of Milano-Bicocca, Monza, Italy

**Keywords:** metabolic myopathies, motor neuron diseases (MNDs), skeletal muscle oxidative metabolism, mitochondrial myopathies, functional evaluation

We are pleased to have the opportunity to edit this Research Topic. We and the other scientists involved in this Research Topic have established a consistent direction toward the possibility of applying the tools of exercise physiology functional evaluation to improve the quality of life (QoL) of frail patients. The goal of this research is the exploration of the complex multifactorial pathogenesis of neuromuscular disorders. The extent of the research ranges from cellular mechanisms to systems derangements and involves pragmatic clinical approaches. We believe that improving the resilience of such patients by addressing exercise intolerance can be a formidable challenge for clinical human physiologists.

Metabolic myopathies (MM) and motor neuron diseases (MND) comprise a heterogeneous group of disorders characterized by impaired oxidative metabolism and reduced muscle strength. Patients affected by these disorders show progressive signs of reduced exercise tolerance and increased fatigability, with devastating effects on their QoL. Taking care of these patients requires clear understanding of those mechanisms which are known to jeopardize oxidative metabolism and skeletal muscle strength during exercise. Especially in MM and MND, exercise triggers inherent flaws in skeletal muscle cellular energy metabolism and motor unit recruitment resulting in severe impairments. Accordingly, the assessment of the impact of precision training and/or diet, “as medicine,” can lead to new therapies aimed at counteracting the effects of these conditions.

It is appropriate that this Research Topic opens with the opinion of Grassi et al.. These authors highlighted the usefulness of widely used, non-invasive physiological approaches to identify and quantify impaired skeletal muscle oxidative metabolism, factors limiting exercise tolerance, and ultimately the patient's QoL.

Questions related to the possible dose-response effect of a precision-based exercise program include:

1) What is the best approach: moderate or high intensity exercise protocol?
2) What is the appropriate duration of rest between exercise sessions?
3) Should aerobic training be performed alone or in combination with strength/balance/flexibility exercises?

If exercise is medicine, these questions are not trivial and change is needed in clinical culture, because the assumption that “exercise is good, exercise is for everybody, so go out and do exercise” is not well-suited for patients with MM and MND. Based on animal model studies, it is observed by Houdebine et al., that in mice with Spinal Muscular Atrophy (SMA), active physical exercise, including high-intensity protocols, induced metabolic adaptations. This finding provides further evidence that exercise in association with gene therapies increases the “survival rate of motor neuron” proteins. Analogous to this, Fiuza-Luces et al., reported the effects of an 8 week training intervention program combining aerobic and resistance exercise in a mouse model with respiratory chain complex I deficiency. It was observed that exercise training increased aerobic fitness and muscle strength, activated signaling pathways involved in muscle mitochondrial biogenesis and anabolism, and improved respiratory chain complexes activity and redox status in muscle tissue.

Physical exercise has been considered harmful for patients with MND and MM for a long time. However, a growing body of evidence now suggests that supervised exercise training is safe and may be effective in improving oxidative capacity and muscle function in patients. MND is a classical paradigm where this issue is fiercely debated. As reported by Tsitkanou et al., the fear of exercise induced damage in motor units and excessive ROS production, leads to the conclusion that accurate dosing of exercise should be considered on an individual basis (see Siciliano et al.). This is a critical issue, since exercise intolerance in MND and functionally similar patients (see Jeppesen et al. for mitochondrial myopathies) will lead to a sedentary life-style (disuse) that, as a vicious cycle, further interferes with QoL. It is noteworthy to mention that Ferri et al. reported that no damage is observed in MND patients doing a precisely-tailored 12-week exercise program. Such evidence is contributing to the body of knowledge that eventually will see the “overwork damage dogma” shattered.

Mohamed et al. shifts the perspective by focusing on training aimed at the sensory portion of muscle control in older patients with MND. The physiological effects of proprioceptive training can possibly decrease the occurrence of muscle fatigue. To our knowledge, a training program aimed at the sensory portion of motor control is a new avenue for further investigation.

The impact of diet on patients with MM and MND have not been deeply explored in this Research Topic. However, Similä et al. reported on the long-term effects of a ketogenic diet in a patient affected by GSD type VII. In the course of a 5-year follow up, an alleviation of muscle symptoms, beneficial effects on breathing, and improvement in exercise performance and oxygen uptake were observed. Similarly, Herrera-Olivares et al. reported on an anecdotal case of a very long-chain acyl-CoA dehydrogenase deficiency, a rare disorder of mitochondrial fatty acid β-oxidation. The patient completed a 6-month supervised aerobic + resistance training program, with the addition of a medium chain triglyceride + carbohydrate supplement ingested 60 min. before each session. Diet and exercise helped to increase the oxidative metabolism safely without muscle contractures or rhabdomyolysis.

One of the challenges in studying exercise in MM and MND is that these diseases are rare and heterogeneous, so it is difficult to recruit a sufficient number of participants for randomized controlled trials, and one type of exercise may not be suitable for everyone.

Therefore, despite the valuable contributions of this research, several points still remain open.

1) Determination of the time, intensity and duration of exercise for different diseases and phenotypes to create a guideline that clinicians and patients can use;
2) Determination of whether continued compliance with nutrition and exercise slows disease progression and minimize muscle deterioration;
3) Most of the data have been obtained in ambulatory patients. The effects of exercise using assistive devices should be further explored in very weak and wheelchair-bound patients;
4) Sometimes patients did not feel any benefit from the exercise nor did the intervention increase their functional capacities. Thus, strategies aimed at empowering these patients and increasing physical function in their habitual tasks should be investigated.

We strongly believe that the future of clinical exercise physiology will be closely linked to the establishment of a new cultural approach among clinicians and researchers about the use of exercise (and diet) as a safe and appropriate way to fight neuromuscular diseases so as to provide suitable care for children and adults.

## Author Contributions

FL wrote the first draft of the present manuscript. LT and MM contributed to its critical revision. All authors contributed to the article and approved the submitted version.

## Conflict of Interest

The authors declare that the research was conducted in the absence of any commercial or financial relationships that could be construed as a potential conflict of interest.

